# Elucidating the Role of Extracellular Vesicles in Pancreatic Cancer

**DOI:** 10.3390/cancers13225669

**Published:** 2021-11-12

**Authors:** Akbar Lulu Marzan, Sarah Elizabeth Stewart

**Affiliations:** Department of Biochemistry and Genetics, La Trobe Institute for Molecular Science, La Trobe University, Melbourne, VIC 3086, Australia; 19572414@students.latrobe.edu.au

**Keywords:** pancreatic cancer, biomarkers, extracellular vesicles, microvesicles, exosomes

## Abstract

**Simple Summary:**

Pancreatic cancer is one of the deadliest cancers worldwide. The chance of surviving more than 5 years after initial diagnosis is less than 10%. This is due to a lack of early diagnostics, where often at the time of initial detection the tumour has already spread to different parts of the body and has developed a propensity to develop drug resistance. Therefore, to tackle this devastating disease, it is necessary to identify the key players responsible for driving pancreatic cancer. Numerous studies have found that small bubble-like packages shed by cancer cells, called extracellular vesicles, play an important role in the progression of the disease. Our knowledge on how extracellular vesicles aid in the progression, spread and chemoresistance of pancreatic cancer is the focus of this review. Of note, these extracellular vesicles may serve as biomarkers for earlier detection of pancreatic cancer and could represent drug targets or drug delivery agents for the treatment of pancreatic cancer.

**Abstract:**

Pancreatic cancer is one of the deadliest cancers worldwide, with a 5-year survival rate of less than 10%. This dismal survival rate can be attributed to several factors including insufficient diagnostics, rapid metastasis and chemoresistance. To identify new treatment options for improved patient outcomes, it is crucial to investigate the underlying mechanisms that contribute to pancreatic cancer progression. Accumulating evidence suggests that extracellular vesicles, including exosomes and microvesicles, are critical players in pancreatic cancer progression and chemoresistance. In addition, extracellular vesicles also have the potential to serve as promising biomarkers, therapeutic targets and drug delivery tools for the treatment of pancreatic cancer. In this review, we aim to summarise the current knowledge on the role of extracellular vesicles in pancreatic cancer progression, metastasis, immunity, metabolic dysfunction and chemoresistance, and discuss their potential roles as biomarkers for early diagnosis and drug delivery vehicles for treatment of pancreatic cancer.

## 1. Introduction

### 1.1. Pancreatic Cancer

Pancreatic cancer (PC) is a highly fatal neoplastic disease. It is considered a major cause of cancer-associated mortality, with an overall 5-year survival rate of less than 10% [1]. Once patients are diagnosed with advanced PC, their life expectancy does not exceed 6 months [2]. The extremely poor prognosis associated with PC can be attributed to a number of factors, including the rapid progression of the disease, high metastatic propensity, lack of early diagnostic symptoms or biomarkers and development of therapeutic resistance to both chemotherapy and radiotherapy [3,4]. Early resection of the tumour is considered to be the only successful treatment regime for this aggressive malignancy. However, by the time of diagnosis, it is metastatic in approximately 80% of the patients, making PC treatment extremely daunting [5,6]. Despite several studies outlining various signalling pathways involved in PC progression, the underlying mechanism behind this malignancy is poorly understood. Therefore, it is necessary to gain further insight into the cellular and molecular mechanisms contributing to PC progression and chemoresistance.

Over the last two decades, the role of extracellular vesicles (EVs) in mediating intercellular communication has come into focus. These nano-sized, membrane-enclosed vesicles are routinely secreted by different cell types, including cancer cells, into their microenvironment [7,8]. Emerging evidence implicating EVs in cancer progression, metastasis and chemoresistance across various malignancies, including PC, have evolved [6,9,10]. In this review, we aim to discuss the current knowledge on the role of EVs in PC progression, metastasis and chemoresistance. In addition, we will discuss their applications as potential biomarkers for early diagnosis and as therapeutic targets and drug delivery vehicles for the treatment of PC.

### 1.2. Extracellular Vesicle Overview

#### 1.2.1. EV Subtypes and Biogenesis

**Figure 1:**
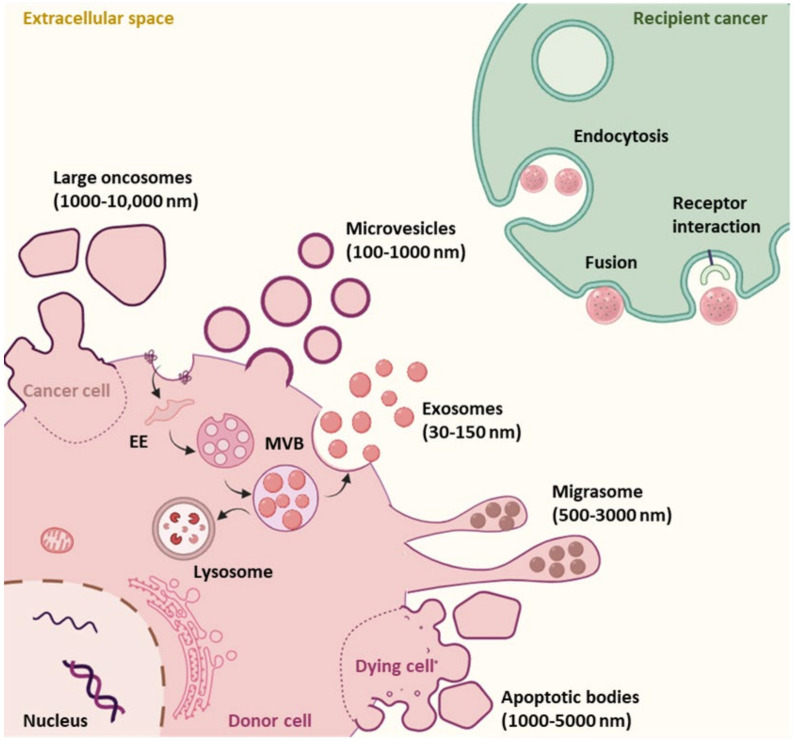
**Schematic representation of extracellular vesicle subtypes, biogenesis and uptake.** Extracellular vesicles (EVs) are classified into different subtypes based on their size and mode of secretion. Exosomes (30–150 nm) are formed within the endosomal system. Maturing from the early endosome to the late endosome, ESCRTs and other related proteins then assist in wrapping recruited cytosolic cargoes with endosomal lipid membrane to form independently closed intraluminal vesicles inside multivesicular bodies (MVBs). MVBs are then either directed toward lysosome for degradation or toward plasma membrane where they fuse to be released from the intraluminal vesicles into extracellular space as exosomes by exocytosis. Microvesicles or ectosomes (100–1000 nm) shed directly from the plasma membrane, encapsulating cytosolic contents. Apoptotic bodies (1000–5000 nm) and large oncosomes (1000–10,000 nm) are secreted from the membrane blebbing of apoptotic cells and cancer cells, respectively. Migrasomes (500–3000 nm) are generated specifically by migrating cells. EVs are transported via bodily fluids to their target recipient cells. EVs may directly fuse with the recipient cell plasma membrane to deliver their contents and can interact to the surface receptors of the target cells or get taken up into the recipient cells through different endocytic mechanisms. Created with https://biorender.com/ (8 October 2021).

EVs are secretory lipid bilayer membrane-bound nanovesicles that can be classified based on their size, biogenesis, and mode of secretion. It is thought that all cells in the human body secrete EVs and they are like a snapshot of the secreting cell, encapsulating active and specific biomolecules from the parent cell. There are several classifications of EVs based on their size and biosynthesis, which include: exosomes (30–150 nm), ectosomes or shedding microvesicles (100–1000 nm), apoptotic bodies (1000–5000 nm), migrasomes (500–3000 nm) and large oncosomes (1000–10,000 nm) (Figure 1) [7,9]. All EVs contain a compendium of functional biomolecules such as proteins, nucleic acids, lipids and metabolites [10,11,12]. Once secreted into the extracellular space, these nanosized particles serve as messengers, delivering their cargo into recipient cells, both near and far from the secreting cell [13,14].

One subtype of particular interest to researchers are exosomes. Exosomes are classified by their biogenesis pathway; they are formed within the endocytic compartment by inward budding within the early–late endosome, resulting in the formation of intraluminal vesicles (ILVs) within the multivesicular body (MVB) (Figure 1). Once formed, these intraluminal vesicles can be released into the extracellular space by exocytosis (via fusion of MVB with the plasma membrane) as exosomes (Figure 1). Alternatively, MVBs can be targeted for degradation and fuse with the lysosome for enzymatic digestion (Figure 1) [15]. However, the exact mechanism behind the sorting and fate of MVBs is poorly understood.

The Endosomal Sorting Complex Required for Transport (ESCRT) machinery containing ESCRT-0, I, II and III are thought to be the major players required for exosome biogenesis. They act in a series to bind, cluster, and sort ubiquitinated proteins into late endosomes [16]. Interestingly, MVBs can also be formed by an ESCRT-independent pathway. This pathway involves the accumulation of sphingomyelin in lipid microdomains (lipid rafts), where the sphingomyelin is converted to ceramide by sphingomyelinases. These ceramide-rich domains are not structurally balanced between lipid monoleaflets, which causes reverse budding of the membrane. This pathway is referred to as the ceramide-dependent pathway [17]. In addition, RAB GTPases and tetraspanins are also involved in the biogenesis and release of exosomes [18,19].

The other major class of EVs of interest are shedding microvesicles (MVs) or ectosomes, which are thought to be larger nanoparticles that are released through outward budding of the plasma membrane (Figure 1) [20,21]. It has been shown that there are several driving factors for MV formation, which include: rearrangement of the actin cytoskeleton and plasma membrane content, accumulation of intracellular calcium, externalization of phosphatidylserine and calpain activation and alterations in local membrane curvature [22,23]. Interestingly, the ESCRT machinery that generates exosomes also participates in MV biogenesis.

Exosomes and MVs are considered to be the major types of EVs secreted by healthy and cancerous cells; however, once secreted into the extracellular space they are very difficult to differentiate from one another. This is due to the fact that their size distribution heavily overlaps with one another, and there are no known protein markers present on one sub-type that are absent on the other. It is thought that exosomes and MVs are differentially enriched in specific proteins that represent the organelles they originate from, which may help in differentiating between these subtypes, but it is not absolute. For instance, exosomes originate from the endocytic pathway and the MVB is thought to be enriched in the tetraspanin cluster of differentiation (CD) CD63, as well as ESCRT components such as tumour susceptibility gene 101 (TSG101), programmed cell death 6-interacting protein (PDCD6IP; also known as ALG-2-interacting protein 1, ALIX) and syntenin [24,25,26,27]. MVs are thought to be enriched in CD9 and CD81 as these tetraspannin are primarily located at the plasma membrane [27,28]. However, CD9 and CD81 are present within the endocytic system (and on MVBs) due to the nature of endocytosis originating from the plasma membrane. Likewise, TSG101 has been reported to be involved in MV shedding and it is unclear whether ALIX and syntenin are specific for exosomes [24,25,26,29]. Therefore, for the purpose of this review, from here on we refer to both exosomes and MVs as EVs as we cannot be sure that they are specifically one subtype or the other and are likely to include a heterogenous population.

Other EV subtypes include apoptotic bodies, migrasomes and large oncosomes (Figure 1). Apoptotic bodies are large EVs secreted from membrane blebs of apoptotic or dying cells [11,30,31]. Migrasomes have open pomegranate-like structures as they harbour multiple small vesicles within their lumen. They are generated at the edge of migrating cells by a process termed as migracytosis [32,33]. Large oncosomes, on the other hand, are released from amoeboid cancer cells and are known to be carriers of oncogenic cargo [34].

#### 1.2.2. Key Methodologies to Isolate and Characterize EVs

As described above, EVs are a heterogeneous population of membrane-bound nanoparticles of diverse size and density that are secreted by cells of all tissues and organs in both healthy and diseased conditions [7,9]. These EVs are packed with a variety of biomolecules that include surface receptors, proteins, lipids, and nucleic acids and analysis of their cargo can provide useful insights into the state of the parental cells, such as in PC [35]. There are a variety of methods that are available and commonly used to isolate EVs for further analysis. Here, we provide a brief summary of some of the most commonly used techniques. EV isolation techniques are based on known physical properties of EVs, such as size, density and surface content. The most commonly utilized conventional EV isolation techniques include: differential ultracentrifugation [36,37], density gradient centrifugation [38,39], ultrafiltration [40], size-exclusive chromatography [41,42] and immunocapture [38]. Each of these methods come with their own advantages and shortcomings, and while these techniques are good for the isolation of whole EVs, it is important to note that overlaps in their physical properties such as size and density make it difficult to separate EV subpopulations.

Differential centrifugation has long been considered the gold standard for EV isolation since their discovery. It utilizes centrifugal force to separate vesicles based on their sedimentation rate. Larger particles such as cells, cell debris, and apoptotic bodies are first sequentially removed by sedimentation with increasing centrifugal force, followed by smaller particles (smaller EVs which are thought to be MVs and exosomes) at much higher centrifugal forces [37,43,44]. While this method is standard for isolating EVs from cell culture or biological fluids, it is thought to be crude and contain protein contaminants. Density gradient centrifugation is another traditional method used for isolation of EVs. This technique separates EVs and contaminants based on their buoyant density into specific layers in solutions of either sucrose, iohexol, or iodixanol [9,38]. Compared to differential centrifugation alone, further purification using a density gradient is thought to yield a purer EV population, containing fewer protein contaminants. This has also long been used for the separation of subcellular components, such as mitochondria, peroxisomes, and endosomes, and the isolation of viral particles [45]. Ultrafiltration is newer in the field but gaining interest as an alternative to differential centrifugation. Ultrafiltration isolates vesicles based on their size by using porous membranes to retain specific size particles and allowing smaller particles to flow through the membranous filter [40,46]. This method is good for concentrating EV samples and usually requires further EV isolation techniques for purification [47]. Size exclusion chromatography is emerging as an excellent method to remove protein contaminants but requires a small/concentrated sample to begin with, which is good for biological fluids such as serum, but it requires a concentration step prior to separation when used for cell culture-derived EVs. During this process, the solution is filtered through a column of porous beads with a radius smaller than the radius of desired EVs [41,42]. Finally, affinity-based techniques such as immuno-capture can be used to isolate EV subpopulations based on the expression of surface receptors. One common example is using antibody-conjugated beads to bind and pull down specific EVs [38,48]. There is now a trend toward the use of several EV isolation techniques together to obtain a purer EV preparation. These techniques are further discussed in detail here [49].

Following the isolation of EVs, a combination of multiple methods is required to characterise EVs and validate the accuracy of isolation and purity of the EVs preparation, in accordance with MISEV guidelines [50]. Transmission electron microscopy and cryo-electron microscopy are used to confirm the morphology and purity of EVs [51]. EV size, concentration and zeta potential can be assessed on the basis of Brownian motion by nanoparticle tracking analysis (NTA) [52] and dynamic light scattering (DLS) [53]. Nanoscale flow cytometry (nanoFACS) is used for the analysis of interested EV populations using antibodies that specifically recognize EVs from heterogeneous population [54]. To further verify the purity of isolated EVs, Western blotting can be used to detect the presence of EV markers such as CD63, CD9, CD81, TSG101 and Alix and rule out contamination with negative controls [55,56].

#### 1.2.3. EV Cell-to-Cell Signalling and Cargo Delivery

Once EVs are secreted into the extracellular space, they are carried via bodil fluids to recipient cells where they interact and deliver their messages to elicit a variety of effects. How EVs elicit their effects on recipient cells varies. In some instances, EVs mediate ligand–receptor interactions at the plasma membrane to stimulate signalling cascades within the recipient cell [57,58]. The other mechanism for mediating an effect in the recipient cell is for the EV to deliver its cargo of active biomolecules into the cytoplasm (or another relevant organelle/compartment). How cargo delivery is achieved remains unclear, and some of the suggested mechanisms for delivery include (1) plasma membrane fusion and (2) via endocytosis (Figure 1). Fusion at the plasma membrane suggests that the EV membrane directly merges with the cell surface membrane. During this process, it is thought that the two lipid bilayer membranes would come in close proximity and form a hemifusion stalk to facilitate the merging of two membranes [59]. Proteins such as Rab, Lamp-1, Sec1/Munc-18 and SNAREs may help with the process of fusion, although it is unclear if they would be available in this instance [60]. Alternatively, EVs may gain entry into the target cell by conjugating with membrane-bound receptors on the recipient cell, not only activating various signalling pathways, but leading to receptor-mediated endocytosis [61]. Both clathrin-mediated and caveolae/caveolin-1-dependent endocytosis have been shown to be crucial players in the uptake of EVs in some instances [62,63]. During clathrin-mediated endocytosis, clathrin-coated vesicles deform the structure of the plasma membrane to form a cave-like structure. Later, these intracellular vesicles un-coat clathrin to fuse with the endosome to unload their cargo [64]. Caveolin-dependent endocytosis, on the other hand, is a lipid raft-mediated endocytosis. Interaction of caveolin-1 with cholesterol results in invagination of the membrane that internalizes EVs into the cell [65]. EVs can also be endocytosed via non-receptor-mediated endocytic pathways, such as micropinocytosis and phagocytosis [63,66,67]. The current knowledge on EV uptake via endocytosis is covered in more detail [68]. Regardless of the uptake mechanism, once in the endocytic system the EVs need to fuse with the limiting endocytic membrane (also known as back-fusion) to deliver their contents. Currently, direct evidence for both fusion at the plasma membrane and fusion within the endocytic compartment is limited but reviewed in detail [69].

## 2. The Role of EVs in Pancreatic Cancer

Cancer patients tend to have more EVs in their circulation compared to healthy individuals [70]. These EVs are enriched with proteins, lipids and nucleic acids, which play a pivotal role in intercellular communication [71]. Several studies have investigated EV-mediated and inferred cargo delivery for the modulation of tumorigenesis, metastasis, immune activation, and therapy resistance in pancreatic cancer, as discussed below (Table 1).

### 2.1. EVs Aid PC Cell Proliferation and Angiogenesis

**Figure 2:**
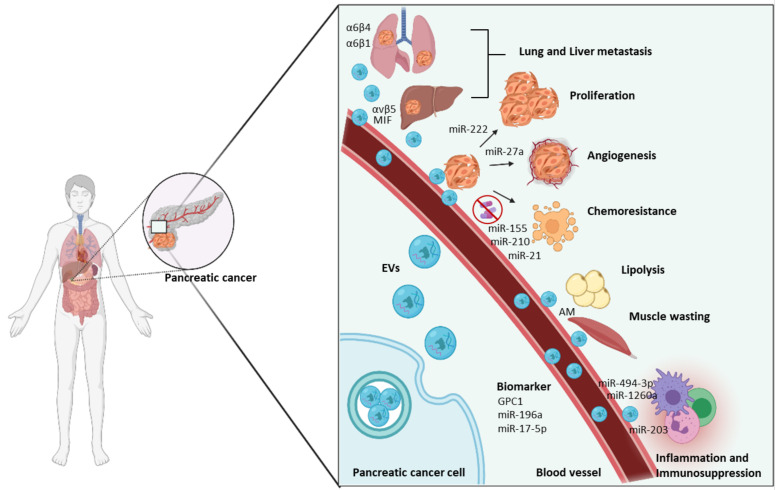
**The role of extracellular vesicles (EVs) in pancreatic cancer (PC).** PC-derived EVs play a vital role in the progression of cancer depending on their content. EVs secreted by PC cells are packed with many biomolecules including miRNAs and proteins which are delivered to recipient cells where they modulate various functions. For instance, PC derived EVs enriched with MIF, α6β4 and α6β1 are involved in metastasis. PC EVs containing miR-222 and miR-27a induces proliferation of cancer cells and angiogenesis. Similarly, EVs packed with miR-155, miR-210, miR-21 participate in the development of chemoresistance and miR-203 helps to escape immunosurveillance. PC EVs also serve as promising diagnostic tool. AM: Adrenomedullin Created with https://biorender.com/ (8 October 2021).

A number of studies have affirmed that EVs function to regulate PC development, progression and angiogenesis. For instance, myoferlin-rich EVs are secreted by PC which stimulate proliferation and migration of PC cells [72,73]. Tumour-derived EVs containing miR-222 were reported to induce PC proliferation and invasion by regulating the expression and re-localization of tumour suppressor P27 (also known as Kip1 or cyclin-dependent kinase inhibitor B). On one hand, miR-222 can directly inhibit the expression of P27; on the other, miR-222 can also phosphorylate p27 via the miR-222/PPP2R2A/AKT pathway that leads to active cytoplasmic p27 expression. Together, these effects contribute to PC proliferation and invasion (Figure 2) [74]. Moreover, in vitro studies by Richards et al. demonstrated that EVs derived from cancer-associated fibroblasts (CAFs), following treatment with gemcitabine, increased the levels of Snail and miR-146a in the recipient PC cell lines to promote proliferation and chemoresistance. Similarly, inhibiting the release of EVs from gemcitabine-treated CAFs reduced proliferation and cell survival [75]. Interestingly, another study found that uptake of CAF-derived annexin-A6-positive EVs by PC cells accelerate PC aggressiveness [76]. In this same study, they found annexin-A6-enriched EVs also aid in PC progression by modulating angiogenesis to supply adequate oxygen and nutrients for the growth of the tumour. Moreover, Shang et al. reported PC cell-derived EVs containing miR-27a stimulate human microvascular endothelial cell proliferation and angiogenesis in PC by reducing the expression of BTG2 [77]. Another study by Novizio and colleagues demonstrated annexin A1-enriched EVs promote PC progression by stimulating the activation of formyl peptide receptors (FPRs) that trigger epithelial–mesenchymal transition of the epithelial cells. This mesenchymal switch is important for stabilizing the neovasculature during angiogenesis [78]. Together, these studies strongly suggest that there is a role for EVs in promoting PC cell proliferation, tumourigenesis and angiogenesis to support growth. Additional studies to further delineate the molecular mechanisms governing these effects will help us fully understand the role of both CAF and PC derived EVs in supporting tumourigenesis.

### 2.2. PC EVs Modulate Invasion and Metastasis

The median survival of patients with PC is less than 6 months [1]. PC is known to mostly metastasize to abdominal sites, especially the liver. Along with the anatomical position of liver that allows entry of pancreatic blood through the hepatic portal vein, the recruitment of PC-derived EVs by the liver makes it an ideal and common metastatic site in PC [6]. Accumulating evidence support the notion that EVs promote the metastatic cascade in PC (Figure 2; Table 1). For instance, one in vivo study showed that 24 h post retro-orbital injection (behind the eye into the retro-orbital venous sinus) fluorescently labelled PC-derived EVs were more efficiently taken up by the liver than lungs and therefore were mostly detected in the liver [79]. Costa-Silva and colleagues have also shown that EVs derived from PC initiate the formation of a pre-metastatic niche in the liver that ultimately increases tumour burden [67] . EVs secreted by PC, enriched with high levels of macrophage migration inhibitory factor (MIF), are readily taken up by the Kupffer cells that alter the liver microenvironment by initiating an inflammatory response and activating fibrotic pathways. These changes assist metastasis in the liver by educating liver cells to recruit immune cells and upregulate the production of TGF-β and fibronectin [67]. Interestingly, PC-derived EVs containing integrin αvβ5 tend to travel to the liver, whereas EVs enriched with α6β4- and α6β1 are primarily found in the lungs (Figure 2) [79]. This specificity of PC EV targeting to different organs, primarily the liver, coincides with known metastatic sites in PC patients and suggest that EVs are important mediators of invasion and metastasis. However, further investigation is essential to understand the molecular mechanisms behind organotrophic metastasis mediated by PC-derived EVs.

### 2.3. PC EVs Promote Chemoresistance in PC

Unfortunately, chemoresistance is a major obstacle in the treatment of PC. Prolonged exposure to gemcitabine, the standard chemotherapeutic agent for PC, results in the development of chemoresistance in the majority of cancer patients [80]. Several recent studies have revealed that EV-mediated cell-to-cell interaction aids the development of drug resistance in multiple cancers by transporting drug resistance-related genes between cells. For instance, it has been shown that proteins such as survivin, [81], Snail [82], P glycoprotein [83] and Multidrug resistant-1 (MDR-1) [84] are trafficked via EVs to recipient cells to promote drug resistance. PC derived EVs have also been reported to deliver a variety of chemoresistance associated miRNAs and proteins to recipient cells to diminish the efficacy of chemotherapeutics, summarised Figure 2 and Table 1. Continuous treatment with gemcitabine has been found to increase the expression of miR-155 in PC cells which subsequently augments the secretion of miR-155 packaged EVs. When miR-155-enriched EVs are taken up by other cancer cells, gemcitabine-associated chemoresistance is increased through the activation of anti-apoptotic pathways and the suppressing gemcitabine-metabolizing enzyme, deoxycytidine kinase [85,86]. Another study showed that EVs derived from gemcitabine-resistant PC stem cells confer drug resistance to gemcitabine-sensitive PC cells by transmitting miR-210 [87]. MiR-21 is another chemoresistance marker found to be elevated in PC EVs that promote drug resistance by binding to apoptotic peptidase-activating factor 1 or activating the PI3K/AKT signalling pathway [88,89,90]. Furthermore, EVs have also been found to induce chemoresistance in PC cells by increasing the expression of superoxide dismutase 2, catalase and reactive oxygen species (ROS) detoxifying genes, thereby stimulating ROS detoxification [86]. Long-term treatment with gemcitabine increases EV secretion and upregulates the level of chemoresistance-inducing factor Snail and miR-106b in both CAFs and CAFs-derived EVs. The uptake of CAFs-derived EV Snail and miR-106b by recipient PC cells facilitates gemcitabine resistance by directly targeting miR-146a and TP53INP1, respectively [62,91]. Taken together, EVs derived from PC cells or other cell types from the tumour microenvironment promote drug resistance by altering genes, RNAs, proteins, and signalling pathways, which limits the therapeutic management of PC.

### 2.4. EVs Regulate Tumour-Associated Immunity

The production of various cytokines and recruitment of immunomodulatory cells such as myeloid-derived suppressor cells (MDSCs), tumour-associated macrophages (TAMs) and Tregs are responsible for making the pancreatic tumour microenvironment immunosuppressive [92,93]. Based on EV cargo and the recipient cells, EVs exhibit opposing roles in PC-associated immunity. PC-derived EVs containing miR-203 and miR-212-3p have been shown to decrease the expression of Toll-like receptor 4 (TLR4) and regulatory factor X-associated protein (RFXAP) in dendritic cells, resulting in immune tolerance [94,95]. Furthermore, EVs secreted by PC cells can also suppress the immune system by reducing the expression of human leukocyte antigen D related (HLA-DR) in monocytes, diminishing the ability of natural killer cells to kill the tumour [96,97]. There is evidence that tumour suppressor SMAD4-deficient PC cells secrete miR-1260a and miR-494-3p-positive EVs. These EVs create an immunosuppressive tumour microenvironment when taken up by myeloid-derived suppressor cells by promoting proliferation and glycolysis (Figure 2) [98]. On the contrary, PC-derived EVs positive for Heat Shock Protein 70 (HSP70) are capable of activating the cytolytic activity of natural killer cells [99]. Circulating EVs are also held responsible for promoting an innate inflammatory response and pancreatitis-associated lung injury by activating nucleotide binding oligomerization domain NOD-like receptor protein 3 (NLRP3) and inducing pyroptosis of alveolar macrophages [100]. Taken together, it is clear there is crosstalk between PC-derived EVs and the immune system; however, the underlying mechanisms are yet to be further explored to clarify the contrasting effects of EVs in tumour-associated immunity in PC.

### 2.5. EVs Participate in PC Metabolic Disfunction

Two common paraneoplastic effects of PC are cachexia, metabolic wasting syndrome, and diabetes [101]. The molecular mechanism behind the onset of PC-associated diabetes and weight loss is poorly understood. A previous study revealed that PC-derived EVs transfer adrenomedullin (AM) into β-cells, which triggers ER stress and defects in the unfolded protein response, which result in the inhibition of insulin secretion [102]. In addition, PC-derived EV AM also promotes lipolysis, hence contributing to PC-associated weight loss. PC-derived EVs enriched in AM bind to AM receptors (ADMRs) on the surface of adipocytes, in turn activating p38 and (ERK)1/2 MAP kinase signalling pathways and promote lipolysis by increasing the expression of phosphorylated hormone-sensitive lipase (p-HSL) and phosphorylated perilipin1, known markers of active lipolysis. Furthermore, blocking of ADMR or p38 and MAPK has been shown to decrease lipolysis, suggesting the loss of body weight in PC is contributed by PC EV-associated AM that results in the loss of adipose tissue. [103]. Yang and colleagues have shown that zinc transporter (ZIP4) also plays a significant role in PC-associated cachexia by promoting muscle wasting and through stimulating RAB27B-mediated HSP70 and HSP90 positive EV release. The secretion of EVs by PC has been shown to be augmented by ZIP4 via the upregulation of RAB27B expression through zinc-sensitive transcription factor CREB. The elevation in RAB27B expression in PC promotes the release of HSP70 and HSP90-positive EVs that promote p38 MAPK-mediated muscle catabolism by activating Toll-like receptor 4 (TLR4) [104]. These studies advocate for the role of EVs in metabolic disfunction in PC, particularly their role in PC-associated cachexia. However, additional investigation is necessary to further elucidate the role of EVs in mediating the metabolic reprogramming of PC.

Overall, these findings revealed various functions of EVs in modulation pancreatic cancer progression, invasion, metastasis, chemoresistance, tumour immunity and PC-associated metabolic disorder.

## 3. Potential Applications of EVs in Diagnosis and Treatment of PC

### 3.1. EVs as Potential Diagnostic and Prognostic Biomarkers

Due to lack of early diagnostic symptoms, screening methods and biomarkers, more than 80% of PC patients are diagnosed with metastatic or locally advanced cancer [105]. Currently, the only biomarker available for PC is CA19-9; however, its low sensitivity and specificity makes it less reliable for early PC screening [2]. Therefore, novel early diagnostic markers are critical to improve the overall survival of PC patients. Since EVs secreted by cancer cells play an important role in disease progression, EVs and their contents have promising potential to serve as highly specific early diagnostic and prognostic biomarkers for PC (Table 1). Moreover, the abundance and stability of EVs in various biological fluids increases their potential of being highly sensitivity in PC diagnosis [106].

**Figure 3:**
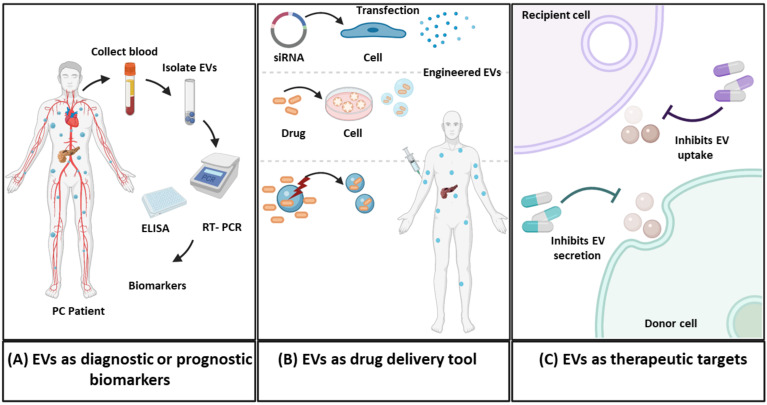
**Potential applications of extracellular vesicles (EVs) in diagnosis and treatment of pancreatic cancer (PC).** (**A**) EVs and their contents have promising potential to serve as highly specific early diagnostic and prognostic biomarkers. Isolating EVs from the plasma of patients and evaluating the expression patterns of miRNA and proteins in EVs could be utilized to discriminate between PC and non-malignant patients. (**B**) EVs can serve as an attractive targeted drug delivery tool for the treatment of PC. As shown, different methods can be used to engineer EVs to carry siRNA, proteins and chemotherapeutics to the recipient cells for treating PC. (**C**) Strategies to block EV release from cancer cells or inhibiting EV uptake by the recipient cells could be a potential therapeutic target for PC. Created with https://biorender.com/ (8 October 2021).

To elucidate whether EVs can be used as biomarkers for PC detection, several groups have isolated EVs from the plasma of PC patients and healthy control individuals and analysed the respective EV miRNA content utilising RT-PCR, a microfluidics-based approach, and localized surface plasmon resonance (LSPR)-based microRNA sensors (Figure 3A). The levels of miR-17-5p, miR-21 [107], miR-550 [108] and miR-10b [109] were found to be upregulated in PC patients, indicating that they may serve as potential biomarkers for the diagnosis of PC (Figure 2). In another study, Xu and colleagues reported high expression of miR-196a and miR-1246 in PC-derived EVs [110]. Madhavan et al. conducted a study where five PC initiating cell markers (EpCAM, CD104, MET, Tspan8 and CD44v6) and four miRNAs (miR-4644 miR-4306, miR-3976, and miR-1246) in serum EVs were analysed. Evaluating the expression levels of PC-initiating cell markers and miRNA serum–EV markers significantly improved the sensitivity and specificity in detecting PC and in differentiating patients with PC from healthy individuals [111].

In addition to miRNA, EV proteins are also sensitive screening tools for PC diagnosis. Melo et al. suggested that glypican-1 (GPC1)-positive EVs secreted by PC could be used as a diagnostic marker for detecting early PC. Mass spectrometry analysis of circulating EVs isolated from 190 PC patients and 100 healthy individuals demonstrated that serum samples of PC patients specifically contain a significantly higher amount of GPC1-positive EVs than normal control individuals, indicating a strong correlation between GPC1-positive EVs and PC (Figure 2). The amount of GPC1-positive EVs present in the circulation is reflective of tumour burden and a decrease in the amount GPC1-positive EVs is correlated with improved survival [112]. It is postulated that EVs containing MIF could be a probable prognostic marker for PC progression and metastasis since MIF was found to be significantly upregulated in EVs isolated from the plasma of a PC mouse model and PC patients compared to the healthy control subjects as determined by mass spectrometry. Furthermore, enzyme-linked immune assay (ELISA) showed MIF to be elevated in EVs isolated from patients with PC with progression of disease post-diagnosis compared to healthy controls. Importantly, MIF was markedly elevated in EVs from stage I PC patients prior to liver metastasis [113]. In another study, Hoshino and colleagues found EVs isolated from PC patients with known liver metastasis have higher levels of integrin αvβ5 compared to patients without metastasis, hinting that αvβ5 could be another potential marker of PC metastasis [79]. Collectively, these findings indicate that evaluating the expression patterns of miRNA and proteins in EVs could be utilized to discriminate between PC and non-malignant patients.

### 3.2. EVs as Drug Delivery Tools

In addition to being carriers of various biomolecules which are readily taken up by recipient cells, EVs are nontoxic and have low immunogenicity. These features make EVs an attractive candidate for targeted delivery of chemotherapeutics. Unlike synthetic nanoparticle drug delivery systems, these membrane-bound nanovesicles can deliver their cargo to recipient cells by avoiding systemic toxicity associated with chemotherapy in off-target tissues, therefore improving overall therapeutic effects [114,115]. Moreover, integrin-associated transmembrane protein CD47 protects EVs against being cleared by monocytes [116]. Pascucci et al. demonstrated that treatment of mesenchymal stromal cells (MSCs) with anticancer drug paclitaxel causes MSCs to package and secrete drug containing EVs that inhibit the proliferation of PC cells in vitro (Figure 3B) [117]. Later Kim et al. showed that EV-loaded paclitaxel can be utilized to treat multidrug-resistant cancer. When drug-resistant cancer cells are treated with EV-encapsulated paclitaxel, the drug accumulated in drug-resistant cancer cells bypassing Pgp mediated drug efflux. This reduces drug elimination which results in increased cytotoxicity and improved therapeutic efficacy in resistant tumours [118]. Furthermore, EVs were successfully employed to transport curcumin to the recipient PC cells to induce cytotoxicity [119]. Apart from chemotherapeutics, EVs can also carry siRNA and proteins to recipient cells. For instance, EVs were engineered to carry siRNA-targeting KRAS^G12D^, a common mutation in PC, resulting in the suppression of PC and improvement of overall survival in mice (Figure 3B) [116]. Likewise, the delivery of survivin blocker, survivin T34A mutant, via EVs to the PC cell line MiaPaCa-2 enhanced its sensitivity to gemcitabine [120]. Collectively, all these studies indicate that EVs are promising novel drug delivery tools for the treatment of PC. However, there are some technical limitations that need to be considered, for instance bulk exosome preparation, effective EV delivery, and target specificity of EVs.

### 3.3. EVs as Potential Therapeutic Targets

Since EVs contribute significantly to PC progression and chemoresistance, strategies to block EV release from cancer cells or inhibiting EV uptake by the recipient cells could be a potential therapeutic target for PC (Figure 3C). Arresting EV secretion from gemcitabine-treated CAFs by GW4869 significantly decreased the survival of PC cells, as it inhibited the transfer of chemoresistance [75]. In addition, a reduction in the number of EVs attenuated gemcitabine resistance induced via miR-155 [85]. Furthermore, blockade of EV MIF prevented the formation of the pre-metastatic niche in the liver and therefore metastasis (Figure 3C) [113]. All these studies suggest EVs could be a good target for treating PC. However, as discussed above, EVs are released by almost all cell types, and cell type-specific blockade of EV secretion or uptake will be a challenge that needs to be considered for EVs to be a suitable therapeutic target.

Taken together, there are promising studies focusing on the application of EVs in the diagnosis and treatment of PC. Nonetheless, they are still in progression and further inquiry in this field will result in the advancement in PC treatment.

## 4. Conclusions

Pancreatic cancer is one of the most lethal and incurable malignancies. Due to the lack of sensitive diagnostic tools, most PC patients are diagnosed with advanced or metastatic cancer which develops chemoresistance. Numerous studies have confirmed that EVs play a pivotal role in PC progression, metastasis, inflammation, chemoresistance and immunosurveillance escape. As EVs are readily available in the circulation and actively participate in cell-to-cell communication by shuttling various biomolecules; therefore, EVs and their content are now being investigated for PC diagnosis and specific biomarkers. EVs are also being explored as novel therapeutic targets for PC. Herein, we endeavoured to summarise the role of EVs in PC along with their potential application in diagnosis and treatment. There are promising data that suggest EVs are suitable for clinical applications; however, additional in-depth research is necessary to explore the molecular mechanisms by which EVs promote PC, which will further open up new possibilities for PC treatment.

## Figures and Tables

**Table 1:** Pancreatic cancer-derived EV content and their role in pancreatic cancer.

Role in PC	EV Content	In Vivo/In Vitro	Diagnostic/ Prognostic Marker	Reference
Proteins				
Reflective of tumour burden	Glypican-1	In vivo	Yes	[54]
Promotes diabetes and lipolysis	Adrenomedullin	In vivo	-	[60,62,63]
Muscle wasting	Zinc transporter ZIP4	In vivo	Yes	[60]
	Adrenomedullin	In vivo	-	[60,62,63]
PC progression and metastasis	Annexin A1	In vitro	Yes	[57]
Liver metastasis	Integrin αvβ5	In vivo	Yes	[61]
Lung metastasis	Integrin α6β4 and α6β1	In vivo	Yes	[61]
Promotes pre-metastatic formation	Macrophage migration inhibitory factor (MIF)	In vivo	Yes	[55]
Induces proliferation, migration and invasion	Myoferlin	In vitro	-	[58,59]
	Annexin A6	In vivo	Yes	[56]
	Zinc transporter ZIP4	In vivo	Yes	[60]
Chemoresistance	Zinc transporter ZIP4	In vivo	Yes	[60]
**miRNA**				
Induces immunosuppression	miR-1260a	In vitro	-	[68]
	miR-494-3p	In vitro	-	[68]
Induces chemoresistance	miR-155	In vivo	-	[69,70]
	miR-210	In vivo	-	[71]
	miR-21	In vivo	Yes	[72]
	miR-106b		-	[43]
Upregulated in PC patients	miR-17-5p	In vivo	Yes	[10]
	miR-550	In vivo	Yes	[73]
	miR-10b	In vivo	Yes	[74]
	miR-196a	In vivo	Yes	[75]
Detected in the serum of PC patients	miR-1246	In vivo	Yes	[76]
	miR-4644	In vivo	Yes	[76]
	miR-4306	In vivo	Yes	[76]
	miR-3976	In vivo	Yes	[76]
Promotes proliferation, invasion and angiogenesis	miR-222	In vivo	-	[64]
	miR-27a	In vivo	-	[65]
Induces immunosurveillance escape	miR-203	In vitro	-	[66]
Induces immune tolerance	miR-212-3p	In vitro	-	[67]

## Data Availability

The data presented in this study are available on request from the corresponding author.
